# A Mini-Review on Lichen-Based Nanoparticles and Their Applications as Antimicrobial Agents

**DOI:** 10.3389/fmicb.2021.633090

**Published:** 2021-03-12

**Authors:** Rohit Rattan, Sudeep Shukla, Bharti Sharma, Mamta Bhat

**Affiliations:** ^1^WWF-India Field Office, Rajouri, India; ^2^Environment Pollution Analysis Lab, Bhiwadi, India; ^3^School of Biosciences and Biotechnology, Baba Ghulam Shah Badshah University, Rajouri, India

**Keywords:** lichens, antimicrobial, nanoparticles, green synthesis, applications

## Abstract

Biological entities such as green plants, fungi, and lichens are now a days persistently explored for the synthesis of nanoparticles. Lichen-based nanoparticles are also becoming increasingly popular owing to their biocompatibility, eco-friendliness, and cost-effectiveness. The lichen-based metal nanomaterials, particularly synthesized using green chemistry approaches, have turned out to be great substitutes to conventional antimicrobial therapies. Many scientific reports established the significant antimicrobial properties exhibited by the lichen nanoparticles. Therefore, the present mini-review summarizes an overview of lichen-based nanomaterials, their synthesis, their applications, and the molecular mechanism of their potential as broad spectrum antimicrobial agents for biomedical applications.

## Introduction

Microbial pathogenesis is the cause of morbidity and mortality of millions across the globe annually. Soon after the discovery of the antibiotics, they were widely considered as an effective remedy against pathogens and rightly remained so, till the emergence of antibiotic resistance among the microorganisms (Martínez, 2008). However, the recent advancements in nanotechnology led to the development of nanoparticles that have established as potent broad-spectrum antimicrobial agents (Wang et al., 2017). The biosynthesis of nanoparticles through green synthesis method involves bioreduction of metals or metal oxide to their elemental forms with size ranging from 1 to 100 nm. Therefore, this process is gaining considerable attention for its eco-friendliness and cost-effectiveness (Mie et al., 2014; Hussain et al., 2016).

Lichens, the composite organisms that result from a symbiotic association between fungi and algae possess several bioactive compounds (Kambar et al., 2014) and as such have been researched thoroughly and are well known for their bioactivity against many pathogens. Lately, researchers have been exploring the possibilities of using lichens for synthesizing nanoparticles and further utilizing them as antimicrobial agents (Mie et al., 2014).

Many reports are available on the synthesis of nanoparticles from different types of lichens, namely, *Parmeliopsis ambigua*, *Punctelia subrudecta*, *Evernia mesomorpha*, and *Xanthoparmelia plitti* (Dasari et al., 2013); *Parmotrema praesorediosum* (Mie et al., 2014); *Cetraria islandica* (Yıldız et al., 2014; Baláž et al., 2020); *Ramalina dumeticola* (Din et al., 2015); *Acroscyphus* sp. and *Sticta* sp. (Debnath et al., 2016); *Parmelia perlata* (Leela and Anchana Devi, 2017); *Usnea longissima* (Siddiqi et al., 2018); *Parmotrema tinctorum* (Khandel et al., 2018); *Parmelia sulcata* (Gandhi et al., 2019); *Protoparmeliopsis muralis* (Alavi et al., 2019); *Ramalina sinensis* (Safarkar et al., 2020); *Cladonia rangiferina* (Devasena et al., 2014; Rai and Gupta, 2019); *Pseudevernia furfuracea* and *Lobaria pulmonaria* (Goga et al., 2020); *Xanthoria elegans*, *Usnea antarctica*, and *Leptogium puberulum* (Baláž et al., 2020); and *Lecanora muralis* (Abdullah et al., 2020).

Nanoparticles derived from metals and their oxides such as silver, gold, titanium, cadmium, iron, zinc, and copper have reportedly been synthesized using many lichens (Mie et al., 2014; Çıplak et al., 2018; Bhat, 2018; Alavi et al., 2019; Gandhi et al., 2019). Many of these lichen-based nanoparticles have been reported to exhibit antimicrobial bioactivity against several bacteria and fungi, which could be attributed to their ability to disintegrate the microbial membrane, oxidation of various cellular components, and generation of hydroxyl radicals (Ruparelia et al., 2008; Marambio-Jones and Hoek, 2010). Therefore, the present review highlights the investigation about the utility of lichens as biological laboratories for the sustainable production of antimicrobial metallic nanoparticles.

## Lichen-Derived Nanoparticles: Methodologies and Approaches

The biosynthesis of lichen-derived nanoparticles is gaining popularity these days: as the process does not involve use of any toxic chemicals, therefore, they can be safely used as pharmaceuticals (Kowalski et al., 2011). Researchers around the globe have been following different methodologies such as biomechanical and chemical solid-state synthesis for the synthesis of lichen-based nanoparticles (Baláž et al., 2020). Mie et al. (2014) reported the synthesis of silver nanoparticles by the reduction of silver nitrate using aqueous extract of the lichen *Parmotrema praesorediosum* as a reductant as well as a stabilizer. Nanoparticles were characterized by using ultraviolet (UV)–visible spectroscopy, electron microscopy, energy-dispersive spectroscopy (EDS), and X-ray diffraction (XRD) technique. The cubic structured nanoparticles exhibited an average particle size of 19 nm. Devasena et al. (2014) synthesized magnesium nanoparticles from *Cladonia rangiferina* with an average size of 23 nm. They used light scattering and UV spectroscopy for characterization of the nanoparticles. Din et al. (2015) successfully synthesized silver nanoparticles by the reduction of silver nitrate with the aqueous extract of the lichen *Ramalina dumeticola*. The synthesis of silver nanoparticles in the solution was confirmed by UV–visible spectroscopy at 433 nm. Their physical appearance was characterized by transmission electron microscopy (TEM) and XRD techniques, revealing a cubic shape with an average size of 13 nm. Debnath et al. (2016) reported the biogenic synthesis of gold nanoparticles from *Acroscyphus* sp. *and Sticta* sp. without the addition of any reducing and stabilizing agent. They were quasi-spherical and prismatic in shapes and characterized by UV–visible, Fourier transform infrared (FT-IR) spectroscopy, powder XRD, and TEM. Çıplak et al. (2018) prepared the lichen-reduced graphene oxide (LrGO) bimetallic nanoparticles nanocomposites (LrGO–AgAu) by using the one-pot approach with *Cetraria islandica*. The characterization of nanoparticles, so formed, was carried out using techniques such as TEM, scanning electron microscopy (SEM), XRD, and FT-IR.

Baláž et al. (2020) reported the solid-state mechanochemical synthesis of silver nanoparticles using lichens *Xanthoria elegans*, *C. islandica*, *Usnea antarctica*, and *Leptogium puberulum.* The method involved milling of lichen sample and silver nitrate together in a pulverisette. The milling process was accompanied by recording of XRD pattern, and after the process of milling was complete, the samples were stored in desiccators, and XRD patterns were recorded. TEM analysis and selected area diffraction (SAD) confirmed the formation of silver nanoparticles. Abdullah et al. (2020) used one-pot green synthesis method for the green synthesis of ZnO/TiO_2_/SiO_2_ and Fe_3_O_4_/SiO_2_ nanoparticle composites using the lichen *Lecanora muralis*. XRD, SEM, EDS, and elemental mapping techniques revealed the fabrication of biosynthesized nanostructure. Safarkar et al. (2020) reported the synthesis of iron oxide nanoparticles from the extract of *Ramalina sinensis* by co-precipitation method. They confirmed the synthesis of nanoparticles by UV spectrophotometer, XRD, FT-IR, and field emission SEM–energy-dispersive X-ray spectrometry (FESEM-EDX). They reported the synthesis of spherical iron oxide nanoparticles with particle size ranging from 31.74 to 53.91 nm, which were observed using FESEM. The visible UV spectra obtained for the iron oxide nanoparticles showed peak in the range of 280–320 nm. The nanoparticles exhibited effective antimicrobial properties against *Staphylococcus aureus* and *Pseudomonas aeruginosa.* Goga et al. (2020) used *Pseudevernia furfuracea* and *Lobaria pulmonaria* to synthesize silver nanoparticles with an average size of 10 nm (while a few reached 100 nm) by using solid-state mechanochemical synthesis.

## Antimicrobial Nature of Lichen-Derived Nanoparticles

Lately, researchers have been making attempts to explore and report antimicrobial properties of the different types of lichen-based nanoparticles (Table 1). Mie et al. (2014) reported the antimicrobial activity of *Parmotrema praesorediosum*-derived silver nanoparticles against eight types of pathogenic bacteria including gram-positive and gram-negative bacteria. Their results showed that silver nanoparticles synthesized using *P. praesorediosum* have significant antibacterial activity against gram-negative bacteria. Siddiqi et al. (2018) reported antibacterial activity of *Usnea longissima*-derived silver nanoparticles against six gram-positive (*Staphylococcus aureus*, *Streptococcus mutans*, *Streptococcus pyrogenes*, *Streptococcus viridans*, *Corynebacterium diphtheriae*, and *Corynebacterium xerosis*) and three gram-negative bacteria (*Escherichia coli*, *Klebsiella pneumoniae*, and *Pseudomonas aeruginosa*). The nanoparticles exhibited significant bioactivity against *E. coli* and *K. pneumoniae*, but *S. mutans*, *C. diphtheriae*, and *P. aeruginosa* displayed resistance against them. Baláž et al. (2020) reported that silver nanoparticles produced using *Xanthoria elegans*, *Cetraria islandica*, *Usnea antarctica*, and *Leptogium puberulum* were excellent antibacterial agents against *E. coli* and *S. aureus*. Alavi et al. (2019) observed that *Protoparmeliopsis muralis*-driven metal (Ag and Cu) and metal oxide (TiO_2_, ZnO, and Fe_3_O_4_) nanoparticles exhibited antibacterial, antibiofilm, antiquorum sensing, and antioxidant abilities against multidrug-resistant bacterium *S. aureus* and reference bacteria *E. coli* and *P. aeruginosa*. Abdullah et al. (2020) examined *Lecanora muralis*-driven nanocomposites of Fe_3_O_4_/SiO_2_ and ZnO/TiO_2_/SiO_2_ for their antimicrobial and antifungal properties and reported that they exhibited good bioactivity against three species of pathogenic bacteria (*S. aureus*, *E. coli*, and *Pseudomonas* spp.) and five species of fungi (*Candida albicans*, *Candida* spp., *Aspergillus flavus*, *Aspergillus niger*, and *Aspergillus terreus*).

**TABLE 1:** Characteristics and antimicrobial activity of Lichen Nanoparticles synthesized by different researchers.

S. No	Lichen	Type of NPs	Shape of NPs	Size of NPs (nm)	Activity exhibited against	References
1.	*Parmotrema pseudotinctorum* and *Ramalina hossei*	Ag NPs	Circular	100	Gram-negative bacteria: 1. *Salmonella typhi*	Kumar et al., 2010

					2. *Escherichia coli*	
2.	*Parmotrema praesorediosum*	Ag NPs	Cubical	19	Gram-positive bacteria:	Mie et al., 2014
					1. *Staphylococcus epidermidis*	
					2. *Staphylococcus aureus*	
					3. *Bacillus subtilis*	
					4. *Streptococcus faecalis*	
					Gram-negative bacteria:	
					1. *Proteus vulgaris*	
					2. *Pseudomonas aeruginosa*	
					3. *Serratia marcescens*	
					4. *Salmonella typhi*	

3.	*Ramalina dumeticola*	Ag NPs	Cubical	13	Gram-positive bacteria:	Din et al., 2015
					1. *Staphylococcus epidermidis*	
					2. *Bacillus subtilis*	
					3. *Streptococcus faecalis*	
					Gram-negative bacteria:	
					1. *Proteus vulgaris*	
					2. *Pseudomonas aeruginosa*	
					3. *Serratia marcescens*	
					4. *Salmonella typhi*	

4.	*Parmotrema clavuliferum*	Ag NPs	Spherical	106	Gram-positive bacteria:	Alqahtani et al., 2017
					1. *Bacillus subtilis*	
					2. *Streptococcus faecalis*	
					3. *Staphylococcus aureus*	
					Gram-negative bacteria:	
					1. *Pseudomonas aeruginosa*	

5.	*Parmelia perlata*	Ag NPs	Spherical	–	Gram-positive bacteria:	Leela and Anchana Devi, 2017
					1. *Staphylococcus aureus*	
					2. *Streptococcus spp.*	
					Gram-negative bacteria:	
					1. *Escherichia coli*	
					2. *Klebsiella pneumoniae*	
					3. *Salmonella spp.*	
					4. *Pseudomonas aeruginosa*	
					Fungi:	
					1. *Aspergillus niger*	
					2. *Candida albicans*	

6.	*Usnea longissima*	Ag NPs	Spherical	9.40–11.23	Gram-positive bacteria:	Siddiqi et al., 2018
					1. *Staphylococcus aureus*	
					2. *Streptococcus mutans*	
					3. *Streptococcus pyrogenes*	
					4. *Streptococcus viridans*	
					5. *Corynebacterium xerosis*	
					6. *Corynebacterium diphtheriae*	
					Gram-negative bacteria:	
					1. *Escherichia coli*	
					2. *Klebsiella pneumoniae*	
					3. *Pseudomonas aeruginosa*	

7.	*Protoparmeliopsis muralis*	Ag NPs Cu NPs	Spherical	Ag NPs – 44.87 Cu NPs – 34.38	Gram-positive bacteria:	Alavi et al., 2019
					1. *Staphylococcus aureus*	
					Gram-negative bacteria:	
					1. *Escherichia coli*	
					2. *Pseudomonas aeruginosa*	

8.	*Heterodermia boryi Parmotrema stuppeum*	Ag NPs	Cubic	27.91–37.21 27.69–36.00	Gram-positive bacteria:	Senthil et al., 2019
					1. *Staphylococcus aureus*	
					2. *Viridans streptococci*	
					Gram-negative bacteria:	
					1. *Acinetobacter baumannii*	
					2. *Escherichia coli*	
					3. *Klebsiella pneumoniae*	
					4. *Pseudomonas aeruginosa*	

9.	*Flavopunctelia flaventior* and *Xanthoria parietina*	Ag NPs	Spherical	69–145	Gram-positive bacteria:	Alqahtani et al., 2020
					1. *Staphylococcus aureus*	
					Gram-negative bacteria:	
					1. *Escherichia coli*	
					2. *Pseudomonas aeruginosa*	

10.	*Xanthoria elegans, Cetraria islandica, Usnea antarctica*, and *Leptogium puberulum*	Ag NPs	Bimodal	5–100	Gram-positive bacteria:	Baláž et al., 2020
					1. *Staphylococcus aureus*	
					Gram-negative bacteria:	
					1. *Escherichia coli*	

11.	*Pseudevernia furfuracea* and *Lobaria pulmonaria*	Ag NPs	Bimodal	Majority smaller – 10 Few reaching – 100	Gram-positive bacteria:	Goga et al., 2020
					1. *Staphylococcus aureus*	
					2. *Listeria monocytogenes*	
					3. *Bacillus cereus*	
					Gram-negative bacteria:	
					1. *Escherichia coli*	
					2. *Pseudomonas aeruginosa*	
					3. *Salmonella enterica*	

12.	*Ramalina sinensis*	FeO NPs	Spherical	31.74 – 53.91	Gram-positive bacteria:	Safarkar et al., 2020
					1. *Staphylococcus aureus*	
					Gram-negative bacteria:	
					1. *Pseudomonas aeruginosa*	

*NPs, Nanoparticles.*

## Molecular Mechanism of Antimicrobial Properties of Lichen-Based Nanoparticles

**FIGURE 1:**
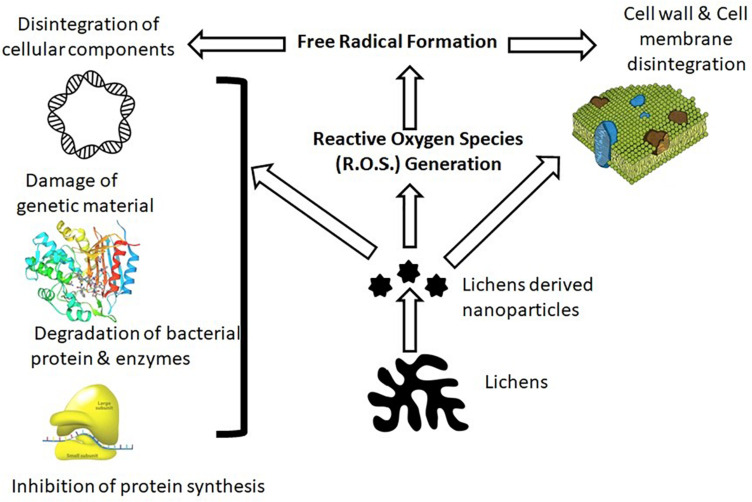
Mechanism of action of lichen nanoparticles on bacteria.

The antimicrobial properties of lichen nanomaterials corroborate their ability to disintegrate microbial cellular barriers (cell wall and membranes), which enable them to penetrate the cytoplasm and disintegrate cellular components and genetic material, which eventually halt their metabolic function (Figure 1; Slavin et al., 2017). However, possible mechanisms of antibacterial activity of lichen nanoparticles have been proposed such as (i) interference during cell wall synthesis, (ii) cellular stress by reactive oxygen species (ROS), (iii) interference in protein synthesis, (iv) disruption of transcription process, (v) disruption of primary metabolic pathways, (vi) inculcation with genetic material, and (vii) alteration in cell signaling process (Dhand et al., 2016). However, studies highlight that the antimicrobial efficacy and molecular mechanism of lichen nanomaterials depend on (i) type of nanomaterial, (ii) shape and size, (iii) microbial membrane composition, and (iv) physicochemical condition (pH, temperature, presence of co-ions, biofilm formation, etc.) (Sánchez-López et al., 2020).

Siddiqi et al. (2018) demonstrated the antimicrobial property of *Usnea longissima*-driven silver nanoparticles through the denaturation of ribosomes that leads to the inactivation of enzymes and proteins, which ultimately stops their metabolic function and results in bacterial apoptosis. Alavi et al. (2019) critically investigated *Protoparmeliopsis muralis* lichen aqueous extract-assisted green synthesis of silver, copper, titanium oxide, zinc oxide, and iron oxide nanoparticles and their associated antibacterial properties. Total antioxidant capacity (TAC) and 2,2-diphenyl-1-picryl-hydrazyl-hydrate (DPPH) antioxidant assay were used to determine the antioxidant property of *P. muralis* lichen. Results clearly indicated that the copper and silver nanoparticles show superior antioxidant and antimicrobial properties over other nanoparticles. Alqahtani et al. (2020) reported that *Xanthoria parietina*- and *Flavopunctelia flaventior*-based silver nanoparticles exhibited greater antibacterial activity against gram-negative bacteria as compared with gram-positive bacteria. This could be attributed to greater penetration of nanoparticles in gram-negative bacteria than that in gram-positive because of a thinner layer of peptidoglycan in the cell wall. Safarkar et al. (2020) reported antimicrobial properties of iron oxide nanoparticle synthesis from *Ramalina sinensis* extract. A study highlights potential antimicrobial efficacy of synthesized nanoparticles against gram-positive and gram-negative bacteria. Electrostatic interaction of positively charged iron nanomaterial and negatively charged bacterial cells may lead to oxidation of bacterial membranes by iron ions, inducing oxidative stress in microbial cells. Production of ROS in stressed microbial cell may further trigger free radical formation. Synthesized free radicals can degenerate various cellular components and may lead to cell death.

## Conclusion

Lichen-mediated nanoparticles are reported as stable, cost-effective, and biocompatible, which make them an ideal candidate for antimicrobial agents. Owing to their unique physical and chemical properties, they exhibit efficacy against a wide spectrum of pathogenic microorganisms such as gram-positive and gram-negative strains of bacteria and some species of fungi. Cost-effectiveness and cellular toxicity are some key concerns that are required to be critically investigated before exploring their antimicrobial candidature widely in pharmaceuticals. The environmental fate of engineered lichen nanomaterials is another big challenge for the sustainable usage of nanotechnology for biological and environmental applications. Therefore, their green synthesis not only can reduce cost of production but also can enhance the associated biocompatibility for living beings.

## Author Contributions

MB prepared the description plan of this review article. RR, SS, and BS carried out the manuscript writing and figure charting. All authors in the manuscript have contributed substantially in the writing of the manuscript and therefore approve it for publication.

## Conflict of Interest

The authors declare that the research was conducted in the absence of any commercial or financial relationships that could be construed as a potential conflict of interest.
